# Evaluation of usability and user feedback to guide telepharmacy application development in Indonesia: a mixed-methods study

**DOI:** 10.1186/s12911-024-02494-3

**Published:** 2024-05-21

**Authors:** Sofa D. Alfian, Jihan A. Sania, Dzulfah Q. Aini, Qisty A. Khoiry, Meliana Griselda, Yudisia Ausi, Neily Zakiyah, Irma M. Puspitasari, Auliya A. Suwantika, Mariska Mahfud, Saktian Aji, Rizky Abdulah, Angelos P. Kassianos

**Affiliations:** 1 Department of Pharmacology and Clinical Pharmacy, Faculty of Pharmacy, Universitas Padjadjaran, Jatinangor, Indonesia; 2 Drug Utilization and Pharmacoepidemiology Research Group, Center of Excellence for Pharmaceutical Care Innovation, Universitas Padjadjaran, Jatinangor, Indonesia; 3 Center for Health Technology Assessment, Universitas Padjadjaran, Jatinangor, Indonesia; 4 Dienggo Kreasi Nusantara Company, Jakarta, Indonesia; 5 Department of Nursing, School of Health Sciences, Cyprus University of Technology, Limassol, Cyprus

**Keywords:** Telemedicine, User-centered design, Mobile application, Indonesia

## Abstract

**Background:**

In Indonesia, the adoption of telepharmacy was propelled by the COVID-19 pandemic, prompting the need for a user-friendly application to support both the general population and pharmacists in accessing healthcare services. Therefore, this study aimed to evaluate usability and user feedback of a pioneering telepharmacy application known as *Tanya Obat* (translating to “Ask about Medications”) in Indonesia, from the perspectives of the general population and pharmacists.

**Methods:**

A mixed-methods sequential study was conducted with the early-stage *Tanya Obat* application in Bandung City. Participants, including the general population and pharmacists, were instructed to use the application for a week. Questionnaires for the general population and pharmacists were distributed from March to May and February to June 2023, respectively. The System Usability Scale questionnaire was adopted to describe usability of the developed application. Further exploration of the quantitative results required collecting open-ended feedback to assess the impressions of the participants, difficulties encountered, and desired features for enhanced user-friendliness. The collected statements were summarized and clustered using thematic analysis. Subsequently, the association between the characteristics of participants and perceived usability was determined with the Chi-square test.

**Result:**

A total of 176 participants, comprising 100 individuals from the general population and 76 pharmacists, engaged in this study. In terms of usability, the questionnaire showed that *Tanya Obat* application was on the borderline of acceptability, with mean scores of 63.4 and 64.1 from the general population and pharmacists, respectively. Additionally, open-ended feedback targeted at achieving a more compelling user experience was categorized into two themes, including concerns regarding the functionality of certain features and recommendations for improved visual aesthetics and bug fixes. No significant associations were observed between the characteristics of participants and perceived usability (p-value > 0.05).

**Conclusion:**

The results showed that the perceived usability of *Tanya Obat* developed for telepharmacy was below average. Therefore, feature optimizations should be performed to facilitate usability of this application in Indonesia.

**Supplementary Information:**

The online version contains supplementary material available at 10.1186/s12911-024-02494-3.

## Background

The development of telepharmacy, with technology incorporation into pharmaceutical care services, was propelled by the COVID-19 pandemic. Telepharmacy provides remote counseling, medication information, online purchases, adverse effects monitoring, and therapy tracking through digital platforms [1, 2]. Moreover, it creates a virtual channel for pharmaceutical care, connecting pharmacists and patients remotely to facilitate easy health evaluation [3]. Other benefits include reducing direct interaction between healthcare professionals and patients, improving pharmaceutical service quality, and minimizing medication errors and adverse effects through various channels such as application, text messaging, video, and voice calls [2]. Telepharmacy improves cost-effectiveness, healthcare access, and after-hours availability, as well as reduces travel time to healthcare facilities [4, 5], breaking geographical barriers and enhancing healthcare accessibility [6, 7]. However, implementing this system in clinical practice may be challenging due to legal considerations, operational costs, and patient trust-building [8, 9].

Recent guidelines in Indonesia mainly address general aspects of delivering telehealth services during the COVID-19 pandemic, particularly focusing on definitions and procedures [10]. Specific regulations for telemedicine are necessary to ensure compliance with ethics and other regulations [11]. Additionally, evaluation of usability and applicability is crucial for optimal implementation and adoption in real-world settings. Gaining insights into usability from the perspectives of the general population and pharmacists during the design and testing stages can enhance application effectiveness, which is essential for successful implementation and scalability [12–14]. Despite the rapid growth of telepharmacy in the country [15–18], only a few available application is tested [19–21], leading to varied usability experiences based on features and limitations encountered [19–21]. Many types of Indonesian telepharmacy application lack appropriate study designs and methods to address engagement issues faced by users and pharmacists. Considering the interconnection between program usability and the engagement of users, usability studies are essential for understanding and improving engagement [22]. Usability is defined as a user interface characteristic that facilitates application adoption, effectiveness, efficiency, and satisfaction in a targeted environment [23]. In telepharmacy implementation, poor usability can hinder technology acceptance [24]. Therefore, the products, systems, processes, and procedures constituting telepharmacy must be designed and implemented to be usable, useful [25], accessible, and user-friendly for both healthcare providers and patients [26]. Engaging these users in usability testing can help to address specific needs and preferences, promoting successful technology acceptance and adoption [27].

An Indonesian telepharmacy application known as *Tanya Obat*, translating to “Ask about Medication”, is developed through design-based study [28] by a team of pharmacists and academicians. This provides a comprehensive ecosystem connecting pharmacies, pharmacists, and technicians with the general population. Furthermore, the application comprises features such as locating nearby pharmacies, online medication purchasing, consultation services, medication use information, and educational resources including webinars, e-modules, and coaching clinics for pharmacists and technicians. *Tanya Obat* differs from other types of application in Indonesia by offering a dedicated ecosystem for pharmacists, including online medication consultation and educational opportunities to enhance competency in delivering health services. Therefore, this study aimed to evaluate *Tanya Obat* usability and obtain user feedback on the application at early development stage in Bandung City from the perspectives of the general population and pharmacists. The association between the characteristics of participants and perceived usability will be explored.

## Method

### Study design

A mixed-methods sequential study design was used to assess usability and obtain user feedback on *Tanya Obat* application at the early development stage before launching. Furthermore, questionnaires for the general population and pharmacists were distributed from March to May and February to June 2023, respectively. Approval with contract number 670/UN6.KEP/EC/2022 was received from the Research Ethics Commission of Universitas Padjadjaran, Indonesia, and all recruited participants provided written informed consent.

### Study population and settings

Participants recruited in Bandung City comprised untrained and first-time *Tanya Obat* users from the general population, irrespective of being health system users, as well as pharmacists registered in the application or not. One week was provided for *Tanya Obat* usage without supervision to permit full exploration of the application, and recruiting was performed online through a convenience sampling method. Inclusion criteria for the general population consisted of individuals with (i) age above 18 years, (ii) ability to use a smartphone, (iii) lack of prior application experience, and (iv) willingness to participate. Meanwhile, the inclusion criteria for pharmacists were (i) current employment at a Community Health Center (CHC), hospital, or pharmacy, (ii) ability to use a smartphone, (iii) no prior experience with the tested application, and (iv) willingness to participate. All participants received an explanation regarding the study stages, application installation, and features.

### *Tanya Obat* application

**Fig. 1:**
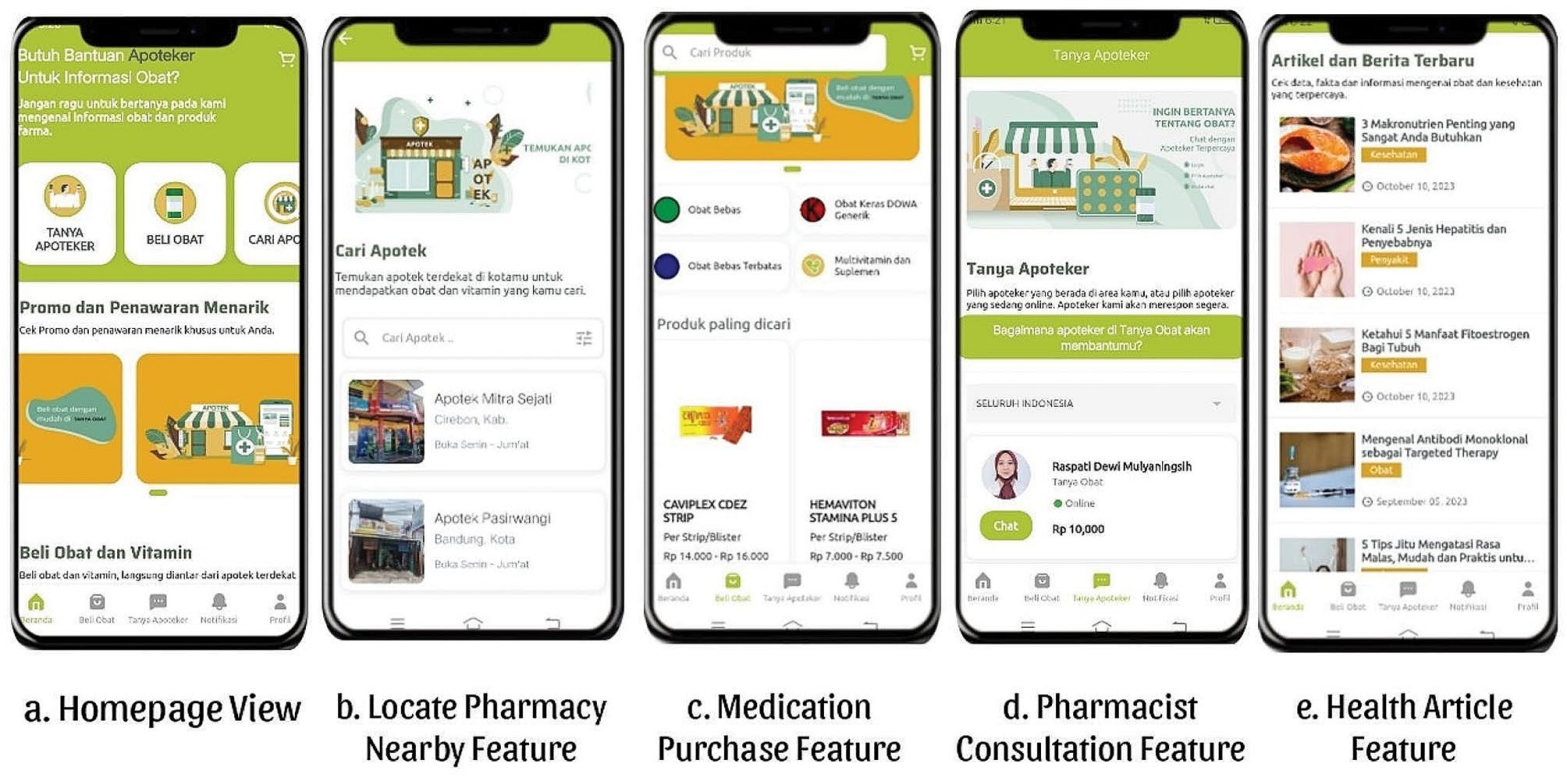
*Tanya Obat* Application Features

*Tanya Obat* application was developed by a team of pharmacists and pharmacy academicians in collaboration with software engineers using Dart (https://dart.dev/) programming language. As shown in Fig. 1, the application offers five main features including:

1. Finding Nearby Pharmacies

This feature allows users to conveniently search for the price sites of nearby pharmacies and view available medication lists, saving time spent on locating the physical buildings.

2. Medication Purchasing

The medication purchasing feature extends the reach of pharmacies to patients through a digital platform. Users can freely browse categories of non-prescription and prescription medications which can be ordered with the delivery service available on the application.

3. Ask a Pharmacist

This flagship feature enables digital consultation with pharmacists regarding self-medication, regular medications currently in use, and other concerns about consumption and side effects. Additionally, it offers pharmacists the opportunity to gain consultation experience, which can subsequently be converted into a credit unit for pursuing professional competency certification. Recruiting for this feature was performed by distributing information through webinars, scientific events, social media and ads, personal and group chat application, as well as direct visits to pharmacies. Users will be charged IDR 20,000 or USD 1.5 for 15-minute consultations based on regulations established by the Indonesian Pharmacists Association in 2019.

4. Health Article

*Tanya Obat* application allows both the general population and pharmacists to access accurate medication information in a clear and easily understandable manner. Moreover, the authors of this study were accredited pharmacists, pharmacy graduates, and students with competencies and excellent knowledge in writing health articles. Each published article was successfully curated and reviewed by professionals and an editorial team.

5. Sustainable Educational Development for Pharmacists

This feature can only be accessed by pharmacists, serving as a channel for enhancing educational knowledge and professional competency through webinars, e-modules, and coaching clinics.

Each feature of *Tanya Obat* application is available for both the general population and pharmacists. However, “Ask a Pharmacist” has slightly different user interfaces tailored for the respective group of participants. This specific feature shows a list of online pharmacists for the general population. Meanwhile, the inability of accounts designed for pharmacists to request consultations as patients, is complemented by the provision of access to current and completed consultation history.

### Measurements

Perceived usability was evaluated based on a standardized quantitative System Usability Scale (SUS). Meanwhile, additional feedback from users engaged in this study was assessed through open-ended qualitative questions.

### Usability measurement

Usability, often defined as user interface characteristic that facilitates application adoption, effectiveness, efficiency, and satisfaction in a targeted environment [23], was assessed with the Indonesian version of the standardized SUS questionnaire [29, 30]. This evaluation was conducted by identifying potential user interface, functionality, and design issues based on the perspective of untrained, first-time users from the general population and pharmacists. Moreover, SUS questionnaire comprised 10 questions rated on a 5-point Likert scale ranging from [1] “Strongly Disagree”, “Disagree”, “Neutral”, “Agree”, to “Strongly Agree” [5]. Participants responded based on the subjective assessment of usability, as presented in Table S1 Supplementary data. Each question contributed a score, with different scoring conversions for odd [1, 3, 5, 7, 9] and even [2, 4, 6, 8, 10] questions, reflecting positive and negative responses, respectively. Summation of all the points yielded a maximum score of 40 which generated a scale ranging from 0 to 100 through multiplication by 2.5. Higher scores suggested favorable user perceptions of the application, while lower scores signified low usability [31]. A previous study showed a significant association between SUS scores reported as continuous or dichotomous data and outcomes [32]. Reporting as dichotomous data may be more practical in real-world scenarios due to the ease of interpretation [33]. Therefore, in this study, a SUS score exceeding 68 points was considered above average in perceived usability [34], calculated based on a published formula [33].

The questionnaire was tested for construct validity and internal consistency using 30 participants who were not included in the main analyses. Construct validity was assessed with the Pearson Product Moment correlation by correlating the score of each question with the total score [35]. The questionnaire was deemed valid when the correlation coefficient (r-value) exceeded the critical value [28]. The values for each question were > 0.361, signifying correlation with the respective dimension and affirming this construct as a measurement instrument. Additionally, Cronbach’s Alpha coefficient yielded a score of 0.721, meeting the criterion established for internal consistency, namely a value > 0.60 [36].

### Additional user feedback

After completing SUS questionnaire, participants provided additional feedback based on open-ended questions to obtain impressions following one week of *Tanya Obat* usage. Three questions were used to assess the perceptions of participants about the application (e.g.,), potential confusion or difficulties encountered (e.g.,), and desired features for enhanced user-friendliness. These included “What was your first impression of this application?”, “What would you say was the most challenging aspect of using this application?”, and “What are your recommendations to improve the application?”. Subsequently, a summary of interpretations made by the study team was sent to all participants for verification.

### Demographic characteristics

The survey collected sociodemographic information about the participants, including factors such as gender (male, female), age, highest education level (junior high school, senior high school, bachelor’s degree, registered pharmacists, master’s degree, doctorate), practice settings, and years of practice (for pharmacists). Additionally, participants provided information on subjective experiences with smartphone application (amateur, beginner, skilled, highly skilled), daily duration of smartphone usage in hours (< 1, 1−2, 3−4, and > 4), and internet accessibility at home (available, unavailable).

### Sample size calculation

The sample size was calculated using Slovin’s formula [37] due to a lack of prior knowledge about outcome distributions. To achieve a 95% confidence interval (CI) and a margin error of 0.15 with a statistical power of 80%, a minimum sample size of 100 for the general population and 64 pharmacists was required.

### Data collection

The link to both the application and survey was disseminated through a digital leaflet across social media, as well as online personal and group chat platforms. This application was available for download on the Google Play store, and data were collected using the Qualtrics platform. To prevent duplicate entries, participants were restricted to filling out the questionnaire only once from a single email address. For pharmacists, a convenient sample was formally or personally recruited through the Indonesian Pharmacists Association network. The estimated time for questionnaire completion was 10 min, while participants were initially reminded that the study aimed to assess user-friendliness of the application and not the ability to use the device correctly. Following one week of exploring the application, participants were immediately instructed to complete the questionnaire to avoid bias introduced by repeated usage and becoming overly familiar with the application. Additionally, screenshots of the features found were requested as evidence of exploring the application.

### Data analysis

Basic demographic characteristics were examined using descriptive statistics, where mean SUS scores were analyzed for each subgroup of the general population and pharmacists. Survey quantitative data were tabulated and presented as frequencies, then a Chi-square test was used to determine the association between the characteristics of participants and perceived usability. Open-ended questions were summarized and clustered with thematic analysis, following steps including familiarization with data, initial code generation, theme development, theme review, theme definition, and conduction of independent qualitative analysis by JAS and DQA using NVivo software version 12. Any disagreements among JAS and DQA were resolved through discussion with a third study team member (SDA). To ensure content analysis reliability, continuous discussion and negotiation regarding the content of keywords, broader concepts, and units of meaning were performed among the team.

## Result

### Quantitative results

#### Demographic characteristics

A total of 176 participants (100 individuals from the general population and 76 pharmacists) engaged in this study. Those from the general population were recruited from 26 districts in Bandung City, majorly comprising 18−25 years old (59%) females (71%) with high school qualifications (71%). Furthermore, most participants were ‘skilled’ at using smartphone application (56%), with over four hours of daily smartphone usage (65%), and found to use Wi-Fi at home (72%), as shown in Table 1.

**Table 1:** The characteristics of participants

Characteristics	General population (*N* = 100)		Pharmacists (*N* = 76)	
	n	%	n	%
**Gender**				
Female	71	71	55	74
Male	29	29	21	28
**Age**				
18−25	59	59	8	11
26−33	12	12	41	54
34−41	7	7	17	22
42−49	11	11	3	4
≥ 50	11	11	7	9
**Educational Level**				
Junior high school	2	2	-	-
Senior high school	71	71	-	-
Bachelor’s degree	26	26	-	-
Registered pharmacists	-		66	87
Master’s degree	1	1	9	12
Doctorate degree	-	-	1	1
**Practice Setting**				
Pharmacy	-	-	60	79
Hospital	-	-	10	13
University	-	-	1	1
Industry	-	-	2	3
Missing	-	-	3	4
**Years of practice**				
1−3	-	-	23	30
4−6	-	-	24	32
> 6	-	-	29	38
Experience in using smartphone application				
Amateur	7	7	5	7
Beginner	18	18	21	28
Skilled	56	56	49	64
Highly skilled	19	19	1	1
**Daily smartphone usage (hours)**				
< 1	4	4	5	7
1−2	11	11	5	7
3−4	20	20	18	24
> 4	65	65	48	63
**Wi-Fi Internet Access**				
Available	72	72	56	74
Unavailable	28	28	20	26

Subsequently, a total of 76 responses were obtained from pharmacists in Bandung City, Indonesia. These majorly included females (73.7%), working in community pharmacies (79.0%), with over six years of job experience (38.2%), considered ‘skilled’ in using mobile application (64.5%), possessing access to the internet (89%), and known to use the smartphone for more than four hours daily (63.2%).

#### Associations between the characteristics of participants and perceived usability

The characteristics of the general population and pharmacists were presented in Tables 2 and 3, respectively. This study identified no significant correlations between the characteristics and perceived application usability (p-value > 0.05).

**Table 2:** Associations between the characteristics of the general population and perceived usability (*N* = 100)

No	Characteristics	Below average in usability (SUS score < 68)	Above average in usability (SUS score ≥ 68)	*p*-value
1	**Gender**			0.841
	Female	48 (71.64)	23 (69.7)	
	Male	19 (28.36)	10 (30.3)	
2	**Age**			0.462
	18−25	42 (62.69)	17 (51.52)	
	26−33	6 (8.96)	6 (18.18)	
	34−41	5 (7.46)	3 (9.09)	
	42−49	8 (11.94)	2 (6.06)	
	≥50	6 (8.96)	5 (15.15)	
3	**Education**			0.125
	Senior High School	51 (76.12)	20 (60.61)	
	Bachelor	14 (20.9)	12 (36.36)	
	Master	0 (0)	1 (3.03)	
	Doctorate	2 (2.99)	0 (0)	
4	**Experience in using smartphone application**			0.112
	Amateur	7 (10.45)	0 (0)	
	Beginner	14 (20.9)	4 (12.12)	
	Skilled	33 (49.25)	23 (69.7)	
	Highly Skilled	13 (19.4)	6 (18.18)	
5	**Smartphone Usage**			0.632
	<1	3 (4.48)	1 (3.03)	
	1−2	9 (13.43)	2 (6.06)	
	3−4	14 (20.9)	6 (18.18)	
	>4	41 (61.19)	24 (72.73)	
6	**Wi-Fi Access**			0.720
	No	18 (26.87)	10 (30.3)	
	Yes	49 (73.13)	23 (69.7)	

**Table 3:** Associations between the characteristics of pharmacists and perceived usability (*N* = 76)

No	Characteristics	Below average in usability (SUS score < 68)	Above average in usability (SUS score ≥ 68)	p-value
1	**Gender**			0.412
	Female	37 (75.51)	18 (66.67)	
	Male	12 (24.49)	9 (33.33)	
2	**Age**			0.563
	18−25	3 (6.12)	5 (18.52)	
	26−33	27 (55.1)	14 (51.85)	
	34−41	12 (24.49)	5 (18.52)	
	42−49	2 (4.08)	1 (3.7)	
	≥ 50	5 (10.2)	2 (7.41)	
3	**Education**			0.747
	Registered Pharmacists	42 (85.71)	24 (88.89)	
	Master	6 (12.24)	3 (11.11)	
	Doctorate	1 (2.04)	0 (0)	
4	**Practice Setting**			0.438
	Pharmacy	38 (77.55)	22 (81.48)	
	Hospital	7 (14.29)	3 (11.11)	
	University	0 (0)	1 (3.7)	
	Industry	1 (2.04)	1 (3.7)	
	Missing	3 (6.12)	0 (0)	
5	**Practice Years**			0.724
	1−3	15 (30.61)	8 (29.63)	
	4−6	14 (28.57)	10 (37.04)	
	>6	20 (40.82)	9 (33.33)	
6	**Experience In Using Smartphone Application**			0.246
	Amateur	4 (8.16)	1 (3.7)	
	Beginner	11 (22.45)	10 (37.04)	
	Skilled	34 (69.39)	15 (55.56)	
	Highly Skilled	0 (0)	1 (3.7)	
7	**Smartphone Usage**			0.210
	<1	4 (8.16)	1 (3.7)	
	1−2	4 (8.16)	1 (3.7)	
	3−4	8 (16.33)	10 (37.04)	
	>4	33 (67.35)	15 (55.56)	
8	**Wi-Fi Access**			0.255
	No	15 (30.61)	5 (18.52)	
	Yes	34 (69.39)	22 (81.48)	

#### Usability results

According to the calculation matrix in Table S2 Supplementary data, the average SUS score of the general population was 63.4, representing a below-average perceived usability. A significant variation in scores was observed, ranging from a high of 87.5 to a low of 30. The average SUS score of pharmacists was 64.1 (Table S3, Supplementary data), which was also below average, with a significant variation in scores ranging from 95 to 42.5.

### Qualitative results

Two themes, including concerns and recommendations, were identified from the responses provided to the open-ended questions. These were further divided into subthemes comprising feature functionality for concerns as well as visual enhancements, feature improvements, system improvements, and other parameters for recommendations, as presented in Table 4 along with examples of supporting quotes.

**Table 4:** General population concerns and recommendations for *Tanya Obat* application (*N* = 100)

No.	Category	Concerns	Frequency (N (%))	Example Quote
1	Functionality of features		68 (68)	
		Not all features functioned optimally, such as the experience of delays or errors.		“If I were to use the application on a daily basis, I would be annoyed by the fact that some features are not functioning optimally.” (Participant 8)
		Required assistance in using features such as medication purchase, redeeming prescribed medicines, and consulting a pharmacist.		“I need somebody to help me that will explain some features such as medication purchase, redeeming prescribed medicines, and consulting a pharmacist.” (Participant 38)
		Some features were difficult to locate.		“Some features were cumbersome and difficult to find because it was the first time.” (Participant 40)
**No.**	**Category**	**Recommendations**		
1	Visual Enhancements		43 (43)	
		Improve user interface to enhance engagement through color, style, and layout.		“The screen needs to be improved as it does not have a good contrast and lay out given resolution and size.” (Participant 15)
		Modify the logo and color scheme of the application to be more appealing.		“I think the logo and its color need to be improved to be more appealing to users.” (Participant 13)
		Redesign the homepage layout and make illustrations in notifications more informative or interactive to prevent monotony.		“Would be better to re-design the lay out and make notifications more interactive. Now it seems monotone.” (Participant 22)
		Opt for darker colors when using white text.		“Please use darker color when using white text. White text with bright color makes it difficult to read.” (Participants 10)
		Implement font size adjustments for both features and articles, considering that article fonts may be too small for some readers.		“Health articles were difficult to read because the font size is too small. I am sure others can’t read as well. Moreover, the small font size in another feature needs to be adjusted.” (Participant 9)
		Adjust font colors for readability and ensure consistency in font style and color.		“It was bothering me because of the inconsistency of font style and color. Please fix this issue.” (Participant 11)
2	Features Improvements		22 (22)	
		Expand the network of partnered pharmacies and pharmacists to cover regions effectively.		“It seems that only a small number of pharmacies and pharmacists have agreed to be partner in this application, but the number of pharmacies and pharmacists need to be increased.” (Participant 23)
	Locate nearby pharmacies		15 (15)	
		Provide additional information about pharmacies, such as opening and closing hours.		“I want to know more information about pharmacies, such their opening and closing hours.” (Participant 17)
		Enhance the filter options with selections for city, neighborhood, district, opening hours, and ratings.		“In order to simplify the search option, please enhance the filter options, for example, city where you live, neighborhood, district, opening hours as well as user testimony using star (ratings).” (Participant 32)
		Include informative notes, such as acceptance of national health insurance or information about adjacent clinics.		“It would be nice if important detail such as whether the pharmacies accept national health insurance or more info regarding adjacent clinics can be added to the application.” (Participant 19)
	Medication purchase		45 (45)	
		Include detailed information about indications, composition, dosage, side effects, suitability for specific demographics, images, alternatives, pricing, shipment origin, and estimated delivery time.		“Users will benefit from detailed info about medication, such as indications, composition, dosage, side effects, suitability for specific demographics, images, alternatives, pricing, origin of shipment, and estimated delivery time.” (Participant 24)
		Incorporate a search feature for recommended medicines based on ailments, along with alternatives.		“I suggest that the application has a feature to look for a recommended medicine based on symptoms or ailments, also with their alternatives.” (Participant 17)
		Integrate filters to compare prices from lowest to highest, and distinguish between generic and patented drugs.		“The filters feature needs to be improved, for example something to compare prices of medicine from lowest to highest, distinguish between generic and patented drugs,….” (Participant 35)
		Add a reminder alarm for medication consumption and recommendations for alternative and complementary medicine.		“Since this is application about medication, I will appreciate it if it also has a reminder alarm for me to take my routine medication. Moreover, feature about recommendations for alternative and complementary medicine would be beneficial for users” (Participant 87)
3	System Improvements		32 (32)	
		Address recurring bugs or errors, particularly issues with loading failures for purchasing medicine, locating nearby pharmacies, and consulting a pharmacist.		“The errors need to be immediately solved, for example with loading failures, since it will increase user’ satisfaction.” (Participant 77)
		Provide a guide for first-time users upon login.		“The easy-to-understand guide is needed to be read before using the application.” (Participant 56)
		Enhance the mapping system to prevent errors such as incorrect medication deliveries.		“The mapping system needs to be very accurate, in order to avoid error in delivering the medication.” (Participant 88)
4	Others		12 (12)	
		Increase the number of articles about medications to discourage inappropriate use and mitigate harmful side effects.		“I recommend that the number of health articles should be increased, particularly about inappropriate medication use and harmful side effects.” (Participant 77)
		Intensify marketing on social media to enhance visibility among potential users.		“The application must be easily found by users, thus, massive marketing on social media is urgently needed.” (Participant 91)
		Expedite the development of the iOS version of the application.		“Since now the application is only available in Play Store, the development of the app as the iOS version need to be hastened to enhance visibility among users.” (Participant 75)

#### User feedback: the general population

Concerns identified for *Tanya Obat* were features such as consulting a pharmacist, searching for online medication purchases, and locating nearby pharmacies. Users reported that the application was not functioning optimally and required assistance using features including purchasing medication, redeeming prescribed medicines, and consulting a pharmacist. Additionally, inconsistencies in color schemes and delays or errors when accessing features within the application were noted (Table 4).

Potential enhancements for the application included creating more visually appealing shows in terms of color combinations, layout, and font styles. Additionally, it was suggested to ensure that features function as intended, providing relevant descriptions for each feature, and enhancing filters. System-related recommendations comprised addressing bugs or errors such as loading failures, adding a user guide, improving maps, and enhancing search speed. Other suggestions included introducing *Tanya Obat* on iOS devices and promoting the application among a wider demographic (Table 4).

#### User feedback: pharmacists

Concerns observed in using *Tanya Obat* application were related to medication purchase, address configuration, and specific hurdles in the registration of pharmacists. Another concern was identified with the “consult a pharmacist” feature, where the need for more assistance was reported (Table 5).

**Table 5:** Concerns and recommendations of pharmacists for *Tanya Obat* application (*N* = 76)

No.	Category	Concerns	Frequency (N (%))	Example Quote
1	Functionality of features	Not all features functioned optimally, such as medication purchase, pharmacy address configuration, pharmacist registration difficulties.	34 (44.7)	“My main concern is that, some features are not work properly, for example when purchasing medication.” (Pharmacist 10)
		Required assistance in using the consult a pharmacist feature.	23 (30.2)	“I was confused, what I was supposed to do on consult a pharmacist feature.” (Pharmacist 21)
**No.**	**Category**	**Recommendations**	**Frequency (N (%))**	**Example Quote**
Feature Improvements				
1	Medication purchase		15 (19.7)	
		Offer a diverse range of medicines, supplements, and vitamins from various brands.		“Not only medicines, but also supplements and vitamins from various brands need to be offered.” (Pharmacist 31)
		Provide information on the availability of medicines in nearby pharmacies.		“Rather than only showing nearby pharmacies, would be better to also show the availability of medicines in those respective pharmacies.” (Pharmacist 27)
		Establish numerous collaborations with retail pharmacies to ensure stock of medicine.		“The partnered pharmacies need to be increased, particularly retail pharmacies to ensure the availability of medicine in the application.” (Pharmacist 45)
		Introduce a feature for delivering pre-ordered medicines through the application.		“I think it would be nice to have a pre-ordered medicine which can be delivered through the application.” (Pharmacist 53)
		Partner with third parties that offer courier or express delivery services.		“When patients need the medication right away, the application needs to be partnered up not only with a standard delivery service, but also the express delivery service.” (Pharmacists 33)
2	Consult with a pharmacist		13 (17.1)	
		Provide specialized consultation services for traditional medicines with experts in the field.		“People also used traditional medicines, thus, the consultation with pharmacist feature need to also provide pharmacists with expertise in traditional medicines too.” (Pharmacist 74)
		Offer specialized consultation services related to cosmetics with pharmacists/cosmeticians.		“For cosmetic products, it would be nice to also offer consultation services with pharmacists who have good knowledge in cosmetics or with cosmeticians.” (Pharmacist 45)
3	Online Continuing Professional Development	Incorporate a new feature in the form of a medical history accessible to pharmacists and claimable as credit points for Continuing Professional Development.	5 (6.6)	“Patients will benefit from a feature showing their medical history which is accessible to pharmacists and claimable as credit points for Continuing Professional Development.” (Pharmacist 67)
4	Education	Provide interactive educational features such as videos about pharmacology.	3 (3.9)	“Health article is already there which is nice, but an interactive educational feature such as video, for example about the effects of medicines on the body (pharmacology), will give more benefits for some people.” (Pharmacist 71)

Several suggestions obtained from feedback in open-ended questions were majorly related to features. Regarding the medication purchase feature, the requirement for expanded stock availability and the incorporation of new functionalities was reiterated. Furthermore, pharmacists stated the need for an online Continuing Professional Development (CPD) feature, which would be incorporated by the Indonesian Pharmacists Association as the issuer of competency certificates. This suggestion was provided to support consistent education through seminars, articles, and learning modules, along with the integration of counseling history or medical records of patients that can be accessed by pharmacists in Indonesia and claimed as credit points for CPD. The feature for registration of pharmacists should be enhanced, while modification of the color palette was recommended to ensure increased visibility and maintain satisfactory show and font choices.

## Discussion

### Principal observations

In this study, usability and user feedback of the innovative *Tanya Obat* application were evaluated. Perceived usability was categorized as below average, with average scores of 63.4 and 64.1 for the general population and pharmacists, respectively. The identified concerns primarily included the functionality of some features, while chances for improvement were observed in the areas of visual, features, and system enhancements. These concerns and recommendations slightly differed between the general population and pharmacists.

From the general population perspective, the below-average score obtained might be attributed to disparities in digital literacy, defined as the ability to acknowledge and use information from various sources presented through computers [38]. A national survey conducted among 10,000 Indonesians showed a digital literacy index of 3.49 in 2021 and 3.54 on a scale of 5 in 2022 [39–41], and despite the improvement, digital literacy in the country remained at a moderate level. Previous results showed that the general population and pharmacy students in Indonesia had a positive perception and were willing to use telepharmacy services [42, 43], providing an opportunity for successful implementation of the application.

Several areas for improvement were identified to enhance and adapt the future application to the understanding and capabilities of users, thereby leading to a more usable and useful system [25 ]. Moreover, primarily existing problems included complications using the features, attributed to unfamiliarity with technology, lack of confidence in using electronic devices, or fear of committing mistakes [39]. A survey reported that 69% of 10,000 Indonesians have not accessed health services through digital platforms [39]. Complications while running the application can be reduced by simplifying the log-in process, reducing required tasks, and showing fewer buttons on the screen [44–55]. Selecting the appropriate design, wording, and development language in a user-centered and participatory design process is crucial and may have an important impact on engagement [56]. These improvements tend to increase satisfaction, which plays a vital role in the implementation and continual use of the application [57, 58]. Therefore, developers must prioritize maximizing the application performance to be more user-friendly [44, 59–64].

The similar marginally acceptable score obtained from the feedback of pharmacists signified that more work was needed for usability improvement, as these professionals were expected to be more exposed to telepharmacy application than the general population. This unsatisfying score could be partly explained by the varying levels of readiness to use telepharmacy application [65]. Pharmacists were forced to implement telehealth services without adequate readiness assessment, particularly in a setting without well-established telehealth services before the COVID-19 pandemic, such as Indonesia [66] Furthermore, the main concerns that require adjustment to enhance application usability and reduce errors include expanding the network of available pharmacies and pharmacists with specialties comprising traditional medication and cosmetics. Specialization among pharmacists has various important benefits such as higher adherence and persistence, better clinical outcomes, monetary benefits for both patients and the healthcare system, and higher patient satisfaction [67]. Therefore, expanding the network of the pharmacy sector to include traditional medication specialists and pharmacist cosmetologists will significantly improve the application.

Recommendations of pharmacists regarding giving details about all medication were consistent with a previous study that reported an increment of adherence among patients after providing simple and brief written medication information [68]. Additionally, offering live interaction during counseling led to care quality improvement. Specific concerns need to be addressed, such as ensuring properly configured camera and audio settings, appropriate light quality, stable internet connection, and readiness to assist patients unfamiliar with the technology [69]. Furthermore, pharmacists recommended interactive video learning for application enhancement. This was consistent with another study that showed effectiveness in interactive video [70, 71], with an 89.7% reported increase in learning outcomes [70]. Interactive virtual content had significant effectiveness compared to the online class group method [71]. Another recommendation was related to visualization enhancement, playing an important role in attracting user attention [44] with a tendency to influence perceived usability even when no differences existed in functionality offered [72]. Users preferred simplicity, showing more graphics than crowded text, and consistent style using combinations of colors [45–47, 73–75]. Consequently, telepharmacy application usability would be optimized with the results of this study before proceeding to evaluate effectiveness because the standards of health professionals need to be met [76].

The characteristics of the general population and pharmacists were found to have no significant association with perceived usability. These results showed that the application could be used for all age groups irrespective of educational background, familiarity with smartphone operation, and internet accessibility level [77]. However, certain studies reported inconsistent results on the association between sociodemographic factors and perceived usability among the general population and pharmacists, respectively [77–79]. Significant associations were previously shown between demographic-related variables and usability [80] from an investigation conducted among participants with years of mobile application experience [80]. The less experienced participants using mobile health application in Indonesia and their subjectivity might lead to under or overestimating the experience had with smartphones, which could clarify the non-significant associations observed in this study [81]. Moreover, some unmeasured factors tended to be associated with perceived usability, such as readiness [81], experience in using mobile health application [80], digital literacy [38], and health literacy [82, 83].

### Implications and future directions

The mixed-methods evaluation conducted in this study and the results represent the first step in optimizing the development and evaluation of *Tanya Obat* application. A previous investigation assessed satisfaction with telehealth application using SUS method and showed improvement from 71.8 to 82.5 following enhancements based on the first round of testing [84]. Therefore, SUS score tends to increase when adequate improvements are provided to *Tanya Obat* application. These results support the importance of incorporating usability studies as part of the digital health intervention design process [85].

Continuous explorations to obtain post-refinement data with a larger sample size from a multicenter study in different provinces in Indonesia are essential to provide more accurate representative results. Additional investigations are required to evaluate the impact of *Tanya Obat* application, particularly among patients with chronic diseases who regularly consume medications, as usability may vary between different clinical groups. This can help identify context-related issues in the future, such as patient adherence, access to healthcare, and satisfaction.

### Strengths and limitations

The strengths of this study include the recruitment of two different subjects, namely the general population and pharmacists, representing potential and relevant end users. Furthermore, the quantitative and qualitative data provided by real-world users are crucial in ensuring the addition of obtained views in the application development to improve usability. The inclusion of real-world participants showed important usability problems and unidentified solutions during the development or expert panel review stages. However, one main limitation of convenience sampling is the risk of bias due to the lack of random selection. Certain groups within the population may be overrepresented or underrepresented due to the inability to control the questionnaire link distribution. This skew might lead to the production of results not accurately representing the entire population. For example, the majority of participants were aged below 50 years and had a moderate to high educational level, both of which were factors increasing the tendency of technological proficiency and willingness to adopt new technology [86, 87]. In future investigations, combination of multiple methods, such as random, stratified, or systematic sampling, can improve the quality and representativeness of the convenience sample, which tends to produce more accurate and reliable results, thereby enhancing generalizability. Furthermore, the subjectivity of respondents may be resulting in under or overestimating their experience in using smart-phones which may lead to the non-significant association with the usability score. There was a challenge in ensuring full exploration of all *Tanya Obat* application features, and the reliance on screenshots from feedback provided by participants without direct verification constituted potential limitations. Future investigations need to conduct a real-time observation or the recording of screen activity as a more objective measurement [88]. The method of this study was limited to user-based usability evaluation, focusing solely on the satisfaction aspect, and not incorporating the think-aloud method [89]. Additionally, expert usability evaluation through heuristic tests [90] were not performed, generating a less comprehensive scope for the results obtained. Despite these limitations, *Tanya Obat* application usability was improved before launching by making changes based on the concerns and recommendations of the target user groups.

## Conclusion

In conclusion, the results showed that the developed *Tanya Obat* application was below average in perceived usability. Therefore, specific feature optimizations should be performed, particularly in terms of visual appeal, features, and system functionality, to improve potential acceptance and usability, facilitating successful adoption in Indonesia.

### Electronic supplementary material

Below is the link to the electronic supplementary material.

Supplementary Material 1

## Data Availability

The datasets used and/or analyzed are available from the corresponding authors upon reasonable request.

## References

[1] Ahmed NJ, Almalki ZS, Alsawadi AH, Alturki AA, Bakarman AH, Almuaddi AM (2023). Knowledge, perceptions, and readiness of Telepharmacy among Hospital pharmacists in Saudi Arabia. Healthcare.

[2] Farid AF, Firdausy AZ, Sulaiman AM, Simangunsong DE, Sulistyani FE (2022). Efektivitas Penggunaan Layanan Telefarmasi Di Era Pandemi COVID-19 Dari Perspektif Masyarakat. Jurnal Farmasi Komunitas.

[3] Ridho A, Alfian SD, van Boven JFM, Levita J, Yalcin EA, Le L (2022). Digital Health Technologies to improve medication adherence and treatment outcomes in patients with tuberculosis: systematic review of Randomized controlled trials. J Med Internet Res.

[4] Baldoni S, Amenta F, Ricci G (2019). Telepharmacy services: Present Status and Future perspectives: a review. Med (B Aires).

[5] Ameri A, Salmanizadeh F, Keshvardoost S, Bahaadinbeigy K (2020). Investigating pharmacists’ views on Telepharmacy: prioritizing Key relationships, barriers, and benefits. J Pharm Technol.

[6] Alfian S.D, Insani W.N, Puspitasari I.M, Wawruch M, Abdulah R (2023). Effectiveness and Process Evaluation of Using Digital Health Technologies in Pharmaceutical Care in Low- and Middle-Income Countries: A Systematic Review of Quantitative and Qualitative Studies.. Telemedicine journal and e-health.

[7] Atmojo JT, Sudaryanto WT, Widiyanto A, Ernawati E, Arradini D, Telemedicine (2020). Cost effectiveness, and patients satisfaction: a systematic review. J Health Policy Manage.

[8] Poudel A, Nissen L. Telepharmacy: a pharmacist’s perspective on the clinical benefits and challenges. Integr Pharm Res Pract. 2016;5:75–82. 10.2147/IPRP.S101685 10.2147/IPRP.S101685 PMC5741040 29354542

[9] Elbeddini A, Yeats A. Pharmacist intervention amid the coronavirus disease 2019 (COVID-19) pandemic: From direct patient care to telemedicine. J Pharm Policy Pract. 2020;13(1):1–4. 10.1186/s40545-020-00229-z. 10.1186/s40545-020-00229-z PMC7251049 32501410

[10] Menteri Kesehatan Republik Indonesia. Pedoman, Pelayanan Kesehatan Melalui Telemedicine Pada Masa Pandemi Corona. Virus Disease 2019 (COVID-19) [Internet]. 2021 [cited 2022 Oct 4]. https://jdih.kemkes.go.id/.

[11] Sulistiyono A, Budiyanti RT, Sriatmi. A regulatory framework for telemedicine in Indonesia. Eubios Journal of Asian and International Bioethics [Internet]. 2019 [cited 2023 Nov 17];29(4). https://philpapers.org/rec/SULARF-3.

[12] Greenhalgh T, Wherton J, Papoutsi C, Lynch J, Hughes G, A’Court C et al. Beyond Adoption: A New Framework for Theorizing and Evaluating Nonadoption, Abandonment, and Challenges to the Scale-Up, Spread, and Sustainability of Health and Care Technologies. J Med Internet Res. 2017 N;19(11). 10.2196/jmir.8775 10.2196/jmir.8775 PMC5688245 29092808

[13] Jones RB, Ashurst EJ, Trappes-Lomax T. Searching for a sustainable process of service user and health professional online discussions to facilitate the implementation of e-health. Health Informatics J. 2016;22(4):948–61. 10.1177/1460458215599024 10.1177/1460458215599024 26324052

[14] Zapata BC, Fernández-Alemán JL, Idri A, Toval A. Empirical studies on usability of mHealth apps: a systematic literature review. J Med Syst. 2015;39(2):1–19. 10.1007/s10916-014-0182-2 10.1007/s10916-014-0182-2 25600193

[15] Fitrina Andiani A, Taruna B, Putra1 W, Khoiri A. The Future of Telemedicine in Indonesia During Covid-19 Pandemic Era: Literature Review. Health Technology Assessment in Action. 2022;6(2). 10.18502/htaa.v6i2.12198

[16] Komalasari R. Telemedicine in Pandemic Times in Indonesia: Healthcare Professional’s Perspective. https://services.igi-global.com/resolvedoi/resolve.aspx?doi=104018/978-1-6684-5499-2.ch008 [Internet]. 1AD Jan 1 [cited 2023 Nov 17];138–53. https://www.igi-global.com/chapter/telemedicine-in-pandemic-times-in-indonesia/314113.

[17] Antarsih NR, Setyawati SP, Ningsih S, Deprizon, Sulaiman E, Pujiastuti N. Telehealth Business Potential in Indonesia. Proceedings of the International Conference on Social, Economics, Business, and Education (ICSEBE 2021). 2022;205:73–8. 10.2991/aebmr.k.220107.015

[18] Dentons. - The Rise of Telemedicine in Indonesia [Internet]. [cited 2023 Oct 31]. https://www.dentons.com/en/insights/articles/2020/july/20/the-rise-of-telemedicine-in-indonesia.

[19] Novrianda D, Herini ES, Haryanti F, Supriyadi E, Lazuardi L. Chemo assist for children mobile health application to manage chemotherapy-related symptoms in acute leukemia in Indonesia: a user-centered design approach. BMC Pediatr. 2023;23(1). 10.1186/s12887-023-04076-0 10.1186/s12887-023-04076-0 PMC10227782 37254039

[20] Rahayu SR, Zainafree I, Merzistya ANA, Cahyati WH, Farida E, Wandastuti AD et al. Development of the SIKRIBO Mobile Health Application for Active Tuberculosis Case Detection in Semarang, Indonesia. Healthc Inform Res. 2022;28(4):297–306. 10.4258/hir.2022.28.4.297 10.4258/hir.2022.28.4.297 PMC9672498 36380427

[21] Fitria N, Idrus L, Putri AR, Sari YO. The usability testing of the integrated electronic healthcare services for diabetes mellitus patients during the pandemic in Indonesia. Digit Health. 2023;9. 10.1177/20552076231173227 10.1177/20552076231173227 PMC10161287 37152237

[22] Nitsch M, Dimopoulos CN, Flaschberger E, Saffran K, Kruger JF, Garlock L et al. A Guided Online and Mobile Self-Help Program for Individuals With Eating Disorders: An Iterative Engagement and Usability Study. J Med Internet Res. 2016;18(1). 10.2196/jmir.4972 10.2196/jmir.4972 PMC4726867 26753539

[23] IEC 62366-1. 2015(en), Medical devices — Part 1: Application of usability engineering to medical devices [Internet]. [cited 2023 Oct 30]. https://www.iso.org/obp/ui/#iso:std:iec:62366:-1:ed-1:v1:en.

[24] Scholtz B, Mahmud I, Ramayah T. Does usability matter? An analysis of the impact of usability on technology acceptance in ERP settings. Interdisciplinary Journal of Information, Knowledge, and Management. 2016;11:309–330. 10.28945/3591

[25] Kayser L, Kushniruk A, Osborne RH, Norgaard O, Turner P. Enhancing the effectiveness of consumer-focused health information technology systems through ehealth literacy: a framework for understanding users’ needs. JMIR Hum Factors. 2015;2(1). 10.2196/humanfactors.3696 10.2196/humanfactors.3696 PMC4797661 27025228

[26] Senjam SS, Manna S, Bascaran C. Smartphones-based assistive technology: accessibility features and apps for people with visual impairment, and its usage, challenges, and usability testing. Clinical Optometry. 2021;13:311–22. 10.2147/OPTO.S336361 10.2147/OPTO.S336361 PMC8636846 34866955

[27] Sripathi V, Sandru V (2013). Effective usability testing-knowledge of user centered design is a key requirement. Int J Emerg Technol Adv Eng [Internet].

[28] Jayatilleke BG, Ranawaka GR, Wijesekera C, Kumarasinha MCB (2019). Development of mobile application through design-based research. Asian Association Open Universities J.

[29] Nugroho HNIA, Ferdiana PI. R. Pengujian Usability Website Menggunakan System Usability Scale. JURNAL IPTEKKOM: Jurnal Ilmu Pengetahuan & Teknologi Informasi. 2015;17(1):31.

[30] Brooke JSUS. A quick and dirty usability scale. Usability Evaluation Ind. 2020;207–12.

[31] Bangor A, Staff T, Kortum P, Miller J, Staff T (2009). Determining what individual SUS scores mean: adding an adjective rating scale. J Usability Stud.

[32] Bloom BM, Pott J, Thomas S, Gaunt DR, Hughes TC. Usability of electronic health record systems in UK EDs. Emerg Med J. 2021;38(6):410–415 10.1136/emermed-2020-210401 10.1136/emermed-2020-210401 PMC8165140 33658268

[33] Sauro J, Lewis JR. Quantifying the User Experience. Quantifying the User Experience [Internet]. 2012 [cited 2023 Nov 20]; 10.1016/C2010-0-65192-3

[34] Sauro J. A practical guide to measuring usability. Should you use 5 or 7 point scales. Denver; 2010. 2–13 p.

[35] e Silva JL, de Sousa Mata M, Câmara SMA. do Céu Clara Costa Í, de Medeiros KS, Cobucci RN, Validity and reliability of the lederman Prenatal Self-Evaluation Questionnaire (PSEQ) in Brazil. BMC Pregnancy Childbirth. 2021;21(1):481 10.1186/s12884-021-03959-3 10.1186/s12884-021-03959-3 PMC8254250 34215199

[36] Firdaus MM (2021). Metodologi Penelitian Kuantitatif: Dilengkapi Analisis Regresi IBM SPSS Statistics Version 26.0.

[37] Almeda JV, Capistrano TG, Sarte GM (2010). Elementary statistics.

[38] Spante M, Hashemi SS, Lundin M, Algers A (2018). Digital competence and digital literacy in higher education research: systematic review of concept use. Cogent Educ.

[39] Kominfo. Status Literasi Digital di Indonesia 2022 [Internet]. 2022 [cited 2023 Oct 19]. https://web.kominfo.go.id/sites/default/files/ReportSurveiStatusLiterasiDigitalIndonesia2022.pdf.

[40] Nurhayati-Wolff H. Digital literacy index in Indonesia from 2020 to 2022, by type [Internet]. 2023 [cited 2023 Oct 19]. https://www.statista.com/statistics/1337349/indonesia-digital-literacy-index-by-type/#statisticContainer.

[41] Harmoko DD (2021). Digital literacy as a solution to improve the quality of Indonesia’s Human resources. Res Dev J Educ.

[42] Alfian SD, Khoiry QA, Andhika A, Pratama M, Pradipta IS, Kristina SA, Zairina E et al. Knowledge, perception, and willingness to provide telepharmacy services among pharmacy students: a multicenter cross-sectional study in Indonesia. BMC Medical Education 2023;23(1):1–9. 10.1186/s12909-023-04790-4. 10.1186/s12909-023-04790-4 PMC10601297 37884985

[43] Tjiptoatmadja NN, Alfian SD. Knowledge, Perception, and Willingness to Use Telepharmacy Among the General Population in Indonesia. Front Public Health. 2022;10. 10.3389/fpubh.2022.825554 10.3389/fpubh.2022.825554 PMC9130580 35646788

[44] Wei Y, Zheng P, Deng H, Wang X, Li X, Fu H (2020). Design features for improving Mobile Health intervention user Engagement: systematic review and thematic analysis. J Med Internet Res.

[45] Perski O, Blandford A, Ubhi HK, West R, Michie S (2017). Smokers’ and drinkers’ choice of smartphone applications and expectations of engagement: a think aloud and interview study. BMC Med Inf Decis Mak.

[46] Lazard AJ, Pikowski J, Horrell L, Ross JC, Noar SM, Sutfin EL (2020). Adolescents’ and young adults’ aesthetics and Functionality preferences for Online Tobacco Education. J Cancer Educ.

[47] Ledel Solem IK, Varsi C, Eide H, Kristjansdottir OB, Mirkovic J, Børøsund E (2019). Patients’ needs and requirements for eHealth Pain Management interventions: qualitative study. J Med Internet Res.

[48] Milward J, Deluca P, Drummond C, Watson R, Dunne J, Kimergård A (2017). Usability testing of the BRANCH Smartphone App designed to reduce Harmful drinking in young adults. JMIR Mhealth Uhealth.

[49] Rabin C, Bock B (2011). Desired features of Smartphone Applications promoting physical activity. Telemedicine e-Health.

[50] Coyne I, Prizeman G, Sheehan A, Malone H, While AE (2016). An e-health intervention to support the transition of young people with long-term illnesses to adult healthcare services: design and early use. Patient Educ Couns.

[51] Peng W, Yuan S, Holtz BE (2016). Exploring the Challenges and Opportunities of Health Mobile Apps for individuals with type 2 diabetes living in Rural communities. Telemedicine e-Health.

[52] Gkatzidou V, Hone K, Sutcliffe L, Gibbs J, Sadiq ST, Szczepura A (2015). User interface design for mobile-based sexual health interventions for young people: design recommendations from a qualitative study on an online Chlamydia clinical care pathway. BMC Med Inf Decis Mak.

[53] Nathalie Lyzwinski L, Caffery L, Bambling M, Edirippulige S (2018). University Students’ perspectives on mindfulness and mHealth: a qualitative exploratory study. Am J Health Educ.

[54] Phillips SM, Courneya KS, Welch WA, Gavin KL, Cottrell A, Nielsen A (2019). Breast cancer survivors’ preferences for mHealth physical activity interventions: findings from a mixed methods study. J Cancer Surviv.

[55] Herbeć A, Perski O, Shahab L, West R (2018). Smokers’ views on Personal Carbon Monoxide Monitors, Associated Apps, and their use: an interview and think-Aloud Study. Int J Environ Res Public Health.

[56] Ludden GDS, Van Rompay TJL, Kelders SM, Van Gemert-Pijnen JEWC. How to increase reach and adherence of web-based interventions: A design research viewpoint. J Med Internet Res. 2015;17(7):e4201. 10.2196/jmir.4201 10.2196/jmir.4201 PMC4526989 26163456

[57] Assael H (1995). Consumers behavior.

[58] Dulkhatif HAT, Warso MM, Pengaruh Kualitas Pelayanan, Kepuasan Pelanggan Dan Lokasi, Terhadap Loyalitas Pelanggan Pada Penyedia Jasa Internet Study Pt Noken Mulia Tama Semarang (2016). J Manag.

[59] Partridge SR, McGeechan K, Hebden L, Balestracci K, Wong AT, Denney-Wilson E (2015). Effectiveness of a mHealth Lifestyle Program With Telephone support (TXT2BFiT) to prevent Unhealthy Weight Gain in Young adults: Randomized Controlled Trial. JMIR Mhealth Uhealth.

[60] Recio-Rodriguez J, Agudo Conde C, Calvo-Aponte M, Gonzalez-Viejo N, Fernandez-Alonso C, Mendizabal-Gallastegui N (2018). The effectiveness of a Smartphone application on modifying the intakes of Macro and micronutrients in Primary Care: a Randomized Controlled Trial. The EVIDENT II study. Nutrients.

[61] Nguyen Thanh V, Guignard R, Lancrenon S, Bertrand C, Delva C, Berlin I (2019). Effectiveness of a fully automated internet-based Smoking Cessation Program: a randomized controlled trial (STAMP). Nicotine Tob Res.

[62] Free C, Phillips G, Galli L, Watson L, Felix L, Edwards P (2013). The effectiveness of Mobile-Health Technology-Based Health Behaviour Change or Disease Management Interventions for Health Care consumers: a systematic review. PLoS Med.

[63] Michie S, Richardson M, Johnston M, Abraham C, Francis J, Hardeman W (2013). The behavior change technique taxonomy (v1) of 93 hierarchically clustered techniques: building an International Consensus for the reporting of Behavior Change interventions. Ann Behav Med.

[64] Schwarzer R (2008). Modeling Health Behavior Change: how to predict and modify the Adoption and Maintenance of Health Behaviors. Appl Psychol.

[65] Farha RA, Gharaibeh L, Alzoubi KH, Alhamad H. Exploring Community Pharmacists’ Perception and Readiness Toward Telepharmacy Implementation in Jordan: A Cross-Sectional Study. Telemed J E Health.. 2023; 30(3): 816-824 10.1089/tmj.2023.0264 10.1089/tmj.2023.0264 37676981

[66] Elawady A, Khalil A, Assaf O, Toure S, Cassidy C (2020). Telemedicine during COVID-19: a survey of Health Care professionals’ perceptions. Monaldi Arch Chest Dis.

[67] Zuckerman AD, Whelchel K, Kozlicki M, Simonyan AR, Donovan JL, Gazda NP (2022). Health-system specialty pharmacy role and outcomes: a review of current literature. Am J Health-System Pharm.

[68] Grime J, Blenkinsopp A, Raynor DK, Pollock K, Knapp P (2007). The role and value of written information for patients about individual medicines: a systematic review. Health Expect.

[69] Barnett N, Jubray B. Remote consultations: how pharmacy teams can practise them successfully. Pharm J. 2020.

[70] Sholikhah R, Krisnawati M, Sudiyono (2019). Effectiveness of the Use of Interactive Video Learning Media in Fashion Technology courses. Adv Social Sci Educ Humanit Res.

[71] Vakilian A, Ranjbar E, Hassanipour M, Ahmadinia H, Hasani H (2022). The effectiveness of virtual interactive video in comparison with online classroom in the stroke topic of theoretical neurology in COVID-19 pandemic. J Educ Health Promot.

[72] Tractinsky N. Aesthetics and Apparent Usability: Empirically Assessing Cultural and Methodological Issues. Proceedings of the ACM SIGCHI Conference on Human factors in computing systems. 10.1145/258549.258626

[73] Crane D, Garnett C, Brown J, West R, Michie S. Factors influencing usability of a smartphone app to reduce excessive alcohol consumption: think aloud and interview studies. Front Public Health. 2017;5. 10.3389/fpubh.2017.00039 10.3389/fpubh.2017.00039 PMC5376568 28421175

[74] Su MC, Chen WC, Liu CY, Jou HJ, Hsiao YC, Tsao LI (2015). The design requirements for an E-Health Management platform: addressing the needs of adolescent girls at high risk of metabolic syndrome. Hu Li Za Zhi.

[75] Peters D, Deady M, Glozier N, Harvey S, Calvo RA (2018). Worker Preferences for a Mental Health App within male-dominated industries: participatory study. JMIR Ment Health.

[76] Johnson CM, Johnson TR, Zhang J. A user-centered framework for redesigning health care interfaces. J Biomed Inform. 2005;38(1):75–87. 10.1016/j.jbi.2004.11.005 10.1016/j.jbi.2004.11.005 15694887

[77] Rezaee R, Asadi S, Yazdani A, Rezvani A, Kazeroon AM. Development, usability and quality evaluation of the resilient mobile application for women with breast cancer. Health Sci Rep. 2022;5(4). 10.1002/hsr2.708 10.1002/hsr2.708 PMC9234476 35782301

[78] Padrini-Andrade L, Balda R, de Areco CX, Bandiera-Paiva KCN, Nunes P, Marba M, Evaluation Of Usability Of A Neonatal Health Information System According To The User’S Perception. STM,. Revista Paulista de Pediatria. 2019;37(1):90–6. 10.1590/1984-0462/;2019;37;1;00019 10.1590/1984-0462/;2019;37;1;00019 PMC6362369 30569950

[79] Fitria N, Idrus L, Putri AR, Sari YO (2023). The usability testing of the integrated electronic healthcare services for diabetes mellitus patients during the pandemic in Indonesia. Digit Health.

[80] Mkpojiogu EOC, Hashim NL, Adamu R. Observed Demographic Differentials in User Perceived Satisfaction on the Usability of Mobile Banking Applications. Knowledge Management International Conference (KMICe). 2016;263–8.

[81] Handayani PW, Indriani R, Pinem AA (2021). Mobile health readiness factors: from the perspectives of mobile health users in Indonesia. Inf Med Unlocked.

[82] Schillinger D (2002). Association of Health Literacy with Diabetes Outcomes. JAMA.

[83] Soemitro DH, Analisis Tingkat Health Literacy Dan Pengetahuan Pasien Hipertensi, Di Puskesmas Kabupaten Malang. Calyptra: Jurnal Ilmiah Mahasiswa Universitas Surabaya. 2014;3(1):1–13.

[84] Immanuel SS, Usability Testing Pada Aplikasi Klikdokter Mobile. Berdasarkan ISO 9241-11. Universitas Diponegoro; 2023.

[85] Horvath KJ, Ecklund AM, Hunt SL, Nelson TF, Toomey TL. Developing Internet-based health interventions: a guide for public health researchers and practitioners. J Med Internet Res. 2015;17(1):e28. 10.2196/jmir.3770 10.2196/jmir.3770 PMC4319079 25650702

[86] Yap YY, Tan SH, Choon SW. Elderly’s intention to use technologies: A systematic literature review. Heliyon. 2022;8(1):e08765 10.1055/s-0040-1714693 10.1016/j.heliyon.2022.e08765 PMC8800037 35128090

[87] Rochmawati E, Kamilah F, Iskandar AC. Acceptance of e-health technology among older people: A qualitative study. Nurs Health Sci. 2022;24(2):437–46. 10.1111/nhs.12939 10.1111/nhs.12939 35297152

[88] Richter Lagha R, Burningham Z, Sauer BC, Leng J, Peters C, Huynh T et al. Usability Testing a Potentially Inappropriate Medication Dashboard: A Core Component of the Dashboard Development Process. Appl Clin Inform. 2020;11(4):528–34. 10.1055/s-0040-1714693 10.1055/s-0040-1714693 PMC7425799 32785904

[89] Cho H, Powell D, Pichon A, Kuhns LM, Garofalo R, Schnall R (2019). Eye-tracking retrospective think-aloud as a novel approach for a usability evaluation. Int J Med Inf.

[90] Wahyuningrum T, Kartiko C, Wardhana AC. Exploring e-Commerce Usability by Heuristic Evaluation as a Compelement of System Usability Scale. In: 2020 International Conference on Advancement in Data Science, E-learning and Information Systems (ICADEIS). 2020. pp. 1–5.

