# Supplementary material for: Evaluation of usability and user feedback to guide telepharmacy application development in Indonesia: a mixed-methods study
Source: BMC Med Inform Decis Mak. 2024 May 21;24:130. doi: 10.1186/s12911-024-02494-3 (PMC11106925; doi:10.1186/s12911-024-02494-3)
Supplement: Supplementary file 1 — Supplementary Material 1 [file 12911_2024_2494_MOESM1_ESM.docx]

**APPENDIX**

**Table S1. System Usability Scale Statements and Scores**

| **No.** | **Statements** | **Response Scale*** | | | | |
| --- | --- | --- | --- | --- | --- | --- |
|  |  | **SD** | **D** | **N** | **A** | **SA** |
| 1 | In the future, I will frequently use Tanya Obat. | 1 | 2 | 3 | 4 | 5 |
| 2 | I find Tanya Obat difficult to use. | 1 | 2 | 3 | 4 | 5 |
| 3 | I find the Tanya Obat application easy to use. | 1 | 2 | 3 | 4 | 5 |
| 4 | I need help from others to use Tanya Obat. | 1 | 2 | 3 | 4 | 5 |
| 5 | I feel that the features in Tanya Obat work as they should. | 1 | 2 | 3 | 4 | 5 |
| 6 | I feel that there are many inconsistencies in Tanya Obat. | 1 | 2 | 3 | 4 | 5 |
| 7 | I believe others will quickly understand how to use Tanya Obat. | 1 | 2 | 3 | 4 | 5 |
| 8 | I find the Tanya Obat application confusing. | 1 | 2 | 3 | 4 | 5 |
| 9 | I do not encounter any obstacles when using Tanya Obat. | 1 | 2 | 3 | 4 | 5 |
| 10 | I need to familiarize myself with Tanya Obat before using it. | 1 | 2 | 3 | 4 | 5 |

*SD: Strongly Disagree, D: Disagree, N: Neutral, A: Agree, SA: Strongly Agree (Sharfina & Santoso, 2016).

**Table S2. The General Population SUS Score Calculation Matrix**

| R | Q1 | Q2 | Q3 | Q4 | Q5 | Q6 | Q7 | Q8 | Q9 | Q10 | Total | | Score |
| --- | --- | --- | --- | --- | --- | --- | --- | --- | --- | --- | --- | --- | --- |
| 1 | 4 | 2 | 5 | 1 | 4 | 2 | 4 | 2 | 3 | 2 | 31 | | 77.5 |
| 2 | 4 | 2 | 4 | 2 | 4 | 2 | 4 | 2 | 4 | 2 | 30 | | 75 |
| 3 | 3 | 2 | 4 | 2 | 3 | 4 | 5 | 2 | 3 | 5 | 23 | | 57.5 |
| 4 | 3 | 2 | 4 | 2 | 4 | 2 | 4 | 2 | 4 | 4 | 27 | | 67.5 |
| 5 | 4 | 2 | 4 | 3 | 3 | 3 | 5 | 2 | 4 | 5 | 25 | | 62.5 |
| 6 | 3 | 1 | 5 | 1 | 2 | 4 | 4 | 2 | 2 | 2 | 26 | | 65 |
| 7 | 4 | 2 | 4 | 3 | 4 | 2 | 4 | 2 | 4 | 3 | 28 | | 70 |
| 8 | 3 | 3 | 4 | 3 | 4 | 3 | 3 | 2 | 4 | 3 | 24 | | 60 |
| 9 | 3 | 2 | 4 | 1 | 3 | 3 | 4 | 2 | 2 | 4 | 24 | | 60 |
| 10 | 3 | 2 | 4 | 2 | 4 | 2 | 4 | 2 | 3 | 2 | 28 | | 70 |
| 11 | 2 | 3 | 4 | 2 | 3 | 4 | 3 | 3 | 2 | 3 | 19 | | 47.5 |
| 12 | 2 | 3 | 3 | 3 | 4 | 4 | 4 | 2 | 3 | 4 | 20 | | 50 |
| 13 | 2 | 4 | 2 | 2 | 2 | 3 | 2 | 5 | 1 | 3 | 12 | | 30 |
| 14 | 2 | 2 | 4 | 2 | 4 | 2 | 4 | 2 | 4 | 4 | 26 | | 65 |
| 15 | 3 | 2 | 4 | 1 | 3 | 3 | 3 | 2 | 3 | 2 | 26 | | 65 |
| 16 | 3 | 2 | 4 | 2 | 4 | 2 | 4 | 2 | 4 | 3 | 28 | | 70 |
| 17 | 3 | 2 | 4 | 1 | 3 | 3 | 5 | 2 | 5 | 2 | 30 | | 75 |
| 18 | 3 | 4 | 4 | 2 | 2 | 4 | 4 | 2 | 2 | 4 | 19 | | 47.5 |
| 19 | 3 | 2 | 4 | 2 | 4 | 2 | 4 | 2 | 4 | 4 | 27 | | 67.5 |
| 20 | 4 | 2 | 4 | 1 | 3 | 3 | 5 | 2 | 2 | 2 | 28 | | 70 |
| 21 | 3 | 3 | 3 | 3 | 3 | 3 | 3 | 3 | 3 | 3 | 20 | | 50 |
| 22 | 3 | 3 | 3 | 3 | 3 | 3 | 3 | 3 | 3 | 3 | 20 | | 50 |
| 23 | 4 | 2 | 4 | 3 | 4 | 3 | 3 | 2 | 3 | 4 | 24 | | 60 |
| 24 | 3 | 3 | 3 | 3 | 4 | 2 | 3 | 3 | 3 | 4 | 21 | | 52.5 |
| 25 | 2 | 2 | 4 | 2 | 3 | 4 | 4 | 2 | 2 | 2 | 23 | | 57.5 |
| 26 | 2 | 2 | 3 | 2 | 2 | 4 | 2 | 4 | 2 | 3 | 16 | | 40 |
| 27 | 2 | 1 | 4 | 3 | 1 | 4 | 4 | 3 | 5 | 2 | 23 | | 57.5 |
| 28 | 5 | 3 | 5 | 2 | 4 | 3 | 5 | 1 | 4 | 2 | 32 | | 80 |
| 29 | 3 | 3 | 3 | 3 | 3 | 3 | 3 | 3 | 3 | 3 | 20 | | 50 |
| 30 | 3 | 2 | 4 | 2 | 4 | 3 | 4 | 2 | 4 | 2 | 28 | | 70 |
| 31 | 3 | 2 | 4 | 2 | 3 | 3 | 4 | 2 | 3 | 1 | 27 | | 67.5 |
| 32 | 4 | 2 | 4 | 2 | 4 | 2 | 4 | 2 | 4 | 2 | 30 | | 75 |
| 33 | 3 | 2 | 4 | 3 | 3 | 3 | 3 | 2 | 4 | 4 | 23 | | 57.5 |
| 34 | 3 | 3 | 3 | 3 | 3 | 3 | 3 | 3 | 3 | 3 | 20 | | 50 |
| 35 | 4 | 2 | 5 | 1 | 4 | 3 | 5 | 1 | 3 | 4 | 30 | | 75 |
| 36 | 4 | 3 | 3 | 3 | 3 | 4 | 4 | 1 | 4 | 3 | 24 | | 60 |
| 37 | 3 | 3 | 3 | 3 | 3 | 3 | 3 | 3 | 3 | 4 | 19 | | 47.5 |
| 38 | 4 | 2 | 5 | 1 | 4 | 2 | 4 | 2 | 5 | 3 | 32 | | 80 |
| 39 | 2 | 2 | 4 | 2 | 2 | 3 | 4 | 2 | 4 | 4 | 23 | | 57.5 |
| 40 | 2 | 2 | 4 | 4 | 2 | 2 | 2 | 2 | 4 | 4 | 20 | | 50 |
| 41 | 4 | 1 | 4 | 4 | 3 | 4 | 3 | 3 | 4 | 3 | 23 | | 57.5 |
| 42 | 4 | 3 | 3 | 2 | 4 | 2 | 3 | 3 | 2 | 5 | 21 | | 52.5 |
| 43 | 3 | 2 | 4 | 3 | 3 | 2 | 4 | 3 | 4 | 4 | 24 | | 60 |
| 44 | 3 | 3 | 3 | 3 | 3 | 3 | 4 | 3 | 3 | 3 | 21 | | 52.5 |
| 45 | 2 | 2 | 4 | 3 | 3 | 1 | 4 | 3 | 3 | 2 | 25 | | 62.5 |
| 46 | 4 | 2 | 4 | 3 | 4 | 2 | 4 | 2 | 4 | 2 | 29 | | 72.5 |
| 47 | 3 | 3 | 4 | 3 | 4 | 2 | 4 | 2 | 3 | 4 | 24 | | 60 |
| 48 | 4 | 2 | 5 | 2 | 5 | 2 | 4 | 2 | 4 | 3 | 31 | | 77.5 |
| 49 | 2 | 1 | 4 | 2 | 5 | 2 | 5 | 2 | 4 | 3 | 30 | | 75 |
| 50 | 5 | 1 | 5 | 2 | 5 | 1 | 5 | 1 | 5 | 5 | 35 | | 87.5 |
| 51 | 3 | 2 | 4 | 2 | 2 | 2 | 4 | 2 | 2 | 4 | 23 | | 57.5 |
| 52 | 4 | 3 | 4 | 2 | 3 | 2 | 3 | 2 | 3 | 3 | 25 | | 62.5 |
| 53 | 4 | 1 | 5 | 2 | 4 | 2 | 5 | 2 | 4 | 2 | 33 | | 82.5 |
| 54 | 4 | 3 | 3 | 3 | 4 | 3 | 5 | 2 | 4 | 5 | 24 | | 60 |
| 55 | 3 | 2 | 4 | 2 | 3 | 2 | 4 | 2 | 2 | 3 | 25 | | 62.5 |
| 56 | 4 | 3 | 4 | 3 | 4 | 3 | 4 | 2 | 4 | 4 | 25 | | 62.5 |
| 57 | 4 | 2 | 4 | 2 | 4 | 3 | 4 | 2 | 4 | 3 | 28 | | 70 |
| 58 | 2 | 2 | 4 | 1 | 2 | 3 | 4 | 2 | 2 | 2 | 24 | | 60 |
| 59 | 3 | 3 | 4 | 2 | 4 | 2 | 3 | 2 | 4 | 4 | 25 | | 62.5 |
| 60 | 5 | 2 | 5 | 3 | 4 | 2 | 5 | 2 | 4 | 4 | 30 | | 75 |
| 61 | 5 | 1 | 5 | 2 | 5 | 2 | 4 | 3 | 5 | 4 | 32 | | 80 |
| 62 | 4 | 2 | 4 | 2 | 4 | 2 | 3 | 2 | 4 | 4 | 27 | | 67.5 |
| 63 | 3 | 2 | 4 | 1 | 4 | 2 | 4 | 2 | 4 | 3 | 29 | | 72.5 |
| 64 | 3 | 3 | 3 | 3 | 3 | 3 | 4 | 3 | 3 | 3 | 21 | | 52.5 |
| 65 | 3 | 3 | 3 | 3 | 3 | 3 | 3 | 3 | 3 | 3 | 20 | | 50 |
| 66 | 3 | 3 | 4 | 3 | 3 | 3 | 3 | 3 | 3 | 3 | 21 | | 52.5 |
| 67 | 3 | 3 | 3 | 3 | 3 | 3 | 3 | 2 | 3 | 3 | 21 | | 52.5 |
| 68 | 1 | 2 | 4 | 2 | 4 | 3 | 4 | 2 | 4 | 4 | 24 | | 60 |
| 69 | 4 | 2 | 4 | 2 | 2 | 3 | 4 | 2 | 2 | 2 | 25 | | 62.5 |
| 70 | 4 | 2 | 4 | 3 | 4 | 2 | 5 | 2 | 4 | 4 | 28 | | 70 |
| 71 | 3 | 4 | 3 | 3 | 4 | 2 | 5 | 2 | 2 | 3 | 23 | | 57.5 |
| 72 | 3 | 4 | 4 | 2 | 3 | 3 | 4 | 3 | 3 | 4 | 21 | | 52.5 |
| 73 | 3 | 3 | 3 | 2 | 3 | 4 | 4 | 2 | 4 | 4 | 22 | | 55 |
| 74 | 4 | 4 | 4 | 2 | 4 | 3 | 4 | 3 | 4 | 4 | 24 | | 60 |
| 75 | 4 | 2 | 4 | 1 | 4 | 3 | 4 | 2 | 4 | 2 | 30 | | 75 |
| 76 | 4 | 2 | 4 | 2 | 4 | 2 | 3 | 2 | 3 | 3 | 27 | | 67.5 |
| 77 | 4 | 2 | 4 | 3 | 4 | 4 | 3 | 3 | 4 | 5 | 22 | | 55 |
| 78 | 3 | 3 | 3 | 3 | 4 | 3 | 3 | 3 | 4 | 4 | 21 | | 52.5 |
| 79 | 4 | 2 | 4 | 2 | 4 | 2 | 4 | 2 | 4 | 2 | 30 | | 75 |
| 80 | 4 | 3 | 3 | 3 | 3 | 3 | 4 | 3 | 3 | 3 | 22 | | 55 |
| 81 | 4 | 2 | 4 | 2 | 4 | 2 | 4 | 2 | 4 | 2 | 30 | | 75 |
| 82 | 3 | 2 | 4 | 1 | 2 | 2 | 3 | 2 | 2 | 4 | 23 | | 57.5 |
| 83 | 3 | 3 | 3 | 4 | 4 | 2 | 3 | 3 | 3 | 4 | 20 | | 50 |
| 84 | 3 | 3 | 4 | 2 | 3 | 3 | 4 | 3 | 4 | 3 | 24 | | 60 |
| 85 | 4 | 3 | 4 | 2 | 3 | 2 | 4 | 2 | 4 | 3 | 27 | | 67.5 |
| 86 | 4 | 2 | 4 | 2 | 4 | 2 | 4 | 2 | 4 | 2 | 30 | | 75 |
| 87 | 3 | 4 | 3 | 4 | 4 | 3 | 4 | 3 | 4 | 4 | 20 | | 50 |
| 88 | 3 | 2 | 4 | 2 | 4 | 2 | 4 | 1 | 5 | 4 | 29 | | 72.5 |
| 89 | 3 | 2 | 4 | 2 | 4 | 3 | 3 | 2 | 4 | 2 | 27 | | 67.5 |
| 90 | 3 | 2 | 4 | 2 | 4 | 2 | 4 | 2 | 4 | 4 | 27 | | 67.5 |
| 91 | 4 | 3 | 3 | 3 | 3 | 2 | 4 | 3 | 4 | 4 | 23 | | 57.5 |
| 92 | 4 | 2 | 4 | 2 | 4 | 3 | 4 | 2 | 4 | 4 | 27 | | 67.5 |
| 93 | 2 | 1 | 5 | 1 | 4 | 2 | 4 | 2 | 4 | 1 | 32 | | 80 |
| 94 | 4 | 3 | 3 | 3 | 4 | 1 | 4 | 1 | 5 | 3 | 29 | | 72.5 |
| 95 | 3 | 3 | 4 | 2 | 4 | 2 | 4 | 2 | 3 | 3 | 26 | | 65 |
| 96 | 3 | 2 | 4 | 2 | 4 | 4 | 3 | 4 | 2 | 3 | 21 | | 52.5 |
| 97 | 5 | 1 | 5 | 2 | 5 | 1 | 5 | 2 | 5 | 5 | 34 | | 85 |
| 98 | 5 | 1 | 5 | 1 | 4 | 1 | 5 | 1 | 5 | 5 | 35 | | 87.5 |
| 99 | 3 | 1 | 5 | 1 | 4 | 2 | 4 | 2 | 2 | 2 | 30 | | 75 |
| 100 | 4 | 2 | 4 | 2 | 3 | 2 | 4 | 1 | 5 | 3 | 30 | | 75 |
| Total SUS | | | | | | | | | | | | 63.375 | |

**Table S3. Pharmacists SUS Score Calculation Matrix**

| R | Q1 | Q2 | Q3 | Q4 | Q5 | Q6 | Q7 | Q8 | Q9 | Q10 | Total | | Score |
| --- | --- | --- | --- | --- | --- | --- | --- | --- | --- | --- | --- | --- | --- |
| 1 | 4 | 2 | 5 | 2 | 5 | 1 | 5 | 2 | 5 | 2 | 35 | | 87.5 |
| 2 | 3 | 2 | 4 | 1 | 3 | 3 | 4 | 1 | 4 | 2 | 29 | | 72.5 |
| 3 | 4 | 2 | 4 | 2 | 4 | 2 | 4 | 2 | 4 | 4 | 28 | | 70 |
| 4 | 3 | 2 | 4 | 2 | 4 | 2 | 4 | 2 | 4 | 4 | 27 | | 67.5 |
| 5 | 1 | 3 | 4 | 3 | 4 | 3 | 4 | 3 | 3 | 3 | 21 | | 52.5 |
| 6 | 4 | 2 | 4 | 2 | 4 | 2 | 4 | 2 | 4 | 3 | 29 | | 72.5 |
| 7 | 3 | 3 | 3 | 3 | 4 | 2 | 4 | 2 | 4 | 3 | 25 | | 62.5 |
| 8 | 3 | 3 | 3 | 4 | 3 | 3 | 2 | 3 | 3 | 4 | 17 | | 42.5 |
| 9 | 5 | 2 | 5 | 2 | 5 | 2 | 4 | 1 | 1 | 4 | 29 | | 72.5 |
| 10 | 3 | 2 | 4 | 3 | 4 | 3 | 3 | 2 | 4 | 4 | 24 | | 60 |
| 11 | 4 | 4 | 3 | 3 | 4 | 3 | 4 | 3 | 4 | 4 | 22 | | 55 |
| 12 | 4 | 2 | 4 | 2 | 4 | 2 | 4 | 2 | 4 | 4 | 28 | | 70 |
| 13 | 4 | 3 | 4 | 3 | 4 | 2 | 4 | 2 | 4 | 4 | 26 | | 65 |
| 14 | 3 | 3 | 4 | 3 | 3 | 3 | 3 | 3 | 3 | 4 | 20 | | 50 |
| 15 | 5 | 1 | 5 | 1 | 4 | 2 | 4 | 2 | 4 | 4 | 32 | | 80 |
| 16 | 3 | 3 | 3 | 3 | 2 | 3 | 3 | 3 | 3 | 2 | 20 | | 50 |
| 17 | 4 | 2 | 4 | 2 | 4 | 2 | 4 | 2 | 4 | 3 | 29 | | 72.5 |
| 18 | 3 | 2 | 4 | 2 | 4 | 3 | 4 | 2 | 3 | 4 | 25 | | 62.5 |
| 19 | 4 | 2 | 5 | 2 | 5 | 1 | 4 | 2 | 4 | 4 | 31 | | 77.5 |
| 20 | 4 | 2 | 4 | 2 | 4 | 2 | 4 | 2 | 4 | 4 | 28 | | 70 |
| 21 | 3 | 3 | 4 | 3 | 3 | 3 | 3 | 3 | 3 | 3 | 21 | | 52.5 |
| 22 | 5 | 2 | 5 | 2 | 3 | 3 | 4 | 2 | 3 | 2 | 29 | | 72.5 |
| 23 | 4 | 2 | 4 | 2 | 4 | 2 | 4 | 2 | 4 | 4 | 28 | | 70 |
| 24 | 4 | 2 | 4 | 2 | 4 | 2 | 4 | 2 | 4 | 4 | 28 | | 70 |
| 25 | 3 | 2 | 4 | 1 | 4 | 2 | 4 | 2 | 4 | 4 | 28 | | 70 |
| 26 | 3 | 2 | 4 | 2 | 4 | 2 | 4 | 2 | 4 | 4 | 27 | | 67.5 |
| 27 | 5 | 2 | 5 | 2 | 5 | 3 | 5 | 2 | 5 | 4 | 32 | | 80 |
| 28 | 5 | 5 | 4 | 2 | 4 | 3 | 4 | 2 | 3 | 2 | 26 | | 65 |
| 29 | 5 | 1 | 5 | 1 | 5 | 1 | 5 | 1 | 5 | 3 | 38 | | 95 |
| 30 | 5 | 1 | 5 | 1 | 5 | 1 | 5 | 1 | 5 | 4 | 37 | | 92.5 |
| 31 | 4 | 2 | 4 | 2 | 4 | 2 | 4 | 2 | 4 | 3 | 29 | | 72.5 |
| 32 | 4 | 2 | 5 | 1 | 4 | 2 | 4 | 2 | 5 | 3 | 32 | | 80 |
| 33 | 4 | 2 | 5 | 4 | 4 | 4 | 4 | 2 | 4 | 4 | 25 | | 62.5 |
| 34 | 3 | 2 | 4 | 3 | 4 | 3 | 4 | 2 | 4 | 4 | 25 | | 62.5 |
| 35 | 4 | 2 | 5 | 2 | 3 | 2 | 4 | 2 | 4 | 4 | 28 | | 70 |
| 36 | 4 | 3 | 3 | 4 | 4 | 3 | 3 | 3 | 3 | 4 | 20 | | 50 |
| 37 | 3 | 3 | 3 | 3 | 3 | 2 | 3 | 3 | 2 | 4 | 19 | | 47.5 |
| 38 | 4 | 2 | 4 | 2 | 4 | 2 | 4 | 2 | 4 | 4 | 28 | | 70 |
| 39 | 4 | 2 | 4 | 2 | 4 | 4 | 2 | 3 | 4 | 3 | 24 | | 60 |
| 40 | 3 | 3 | 4 | 2 | 3 | 4 | 4 | 2 | 4 | 4 | 23 | | 57.5 |
| 41 | 3 | 1 | 2 | 2 | 4 | 3 | 3 | 3 | 3 | 3 | 23 | | 57.5 |
| 42 | 4 | 3 | 4 | 2 | 5 | 3 | 4 | 3 | 4 | 3 | 27 | | 67.5 |
| 43 | 3 | 3 | 3 | 3 | 3 | 3 | 3 | 3 | 3 | 3 | 20 | | 50 |
| 44 | 4 | 2 | 4 | 2 | 4 | 2 | 3 | 2 | 3 | 4 | 26 | | 65 |
| 45 | 3 | 3 | 4 | 3 | 4 | 4 | 4 | 3 | 3 | 4 | 21 | | 52.5 |
| 46 | 4 | 3 | 4 | 2 | 3 | 4 | 4 | 2 | 3 | 2 | 25 | | 62.5 |
| 47 | 4 | 2 | 4 | 3 | 2 | 2 | 4 | 3 | 2 | 4 | 22 | | 55 |
| 48 | 4 | 2 | 4 | 1 | 4 | 3 | 4 | 2 | 4 | 2 | 30 | | 75 |
| 49 | 4 | 3 | 3 | 2 | 3 | 3 | 4 | 2 | 4 | 4 | 24 | | 60 |
| 50 | 3 | 2 | 4 | 2 | 3 | 3 | 4 | 2 | 3 | 3 | 25 | | 62.5 |
| 51 | 3 | 3 | 4 | 3 | 4 | 3 | 4 | 2 | 4 | 3 | 25 | | 62.5 |
| 52 | 3 | 2 | 4 | 3 | 4 | 4 | 3 | 2 | 2 | 4 | 21 | | 52.5 |
| 53 | 5 | 2 | 5 | 2 | 4 | 3 | 4 | 2 | 5 | 4 | 30 | | 75 |
| 54 | 4 | 2 | 4 | 2 | 4 | 3 | 4 | 2 | 4 | 4 | 27 | | 67.5 |
| 55 | 4 | 2 | 4 | 2 | 4 | 3 | 4 | 2 | 3 | 3 | 27 | | 67.5 |
| 56 | 4 | 4 | 3 | 2 | 2 | 4 | 3 | 2 | 2 | 4 | 18 | | 45 |
| 57 | 4 | 3 | 3 | 3 | 4 | 3 | 3 | 3 | 3 | 4 | 21 | | 52.5 |
| 58 | 4 | 2 | 4 | 2 | 4 | 4 | 4 | 2 | 4 | 3 | 27 | | 67.5 |
| 59 | 2 | 3 | 5 | 1 | 4 | 5 | 3 | 2 | 4 | 4 | 23 | | 57.5 |
| 60 | 4 | 2 | 3 | 2 | 4 | 2 | 3 | 2 | 4 | 3 | 27 | | 67.5 |
| 61 | 4 | 3 | 3 | 4 | 3 | 3 | 3 | 4 | 3 | 4 | 18 | | 45 |
| 62 | 4 | 2 | 4 | 2 | 4 | 3 | 4 | 2 | 4 | 3 | 28 | | 70 |
| 63 | 4 | 3 | 4 | 2 | 4 | 3 | 4 | 3 | 3 | 3 | 25 | | 62.5 |
| 64 | 4 | 3 | 3 | 3 | 3 | 3 | 3 | 3 | 3 | 3 | 21 | | 52.5 |
| 65 | 3 | 2 | 4 | 2 | 2 | 4 | 4 | 2 | 2 | 4 | 21 | | 52.5 |
| 66 | 3 | 3 | 3 | 2 | 4 | 3 | 3 | 3 | 3 | 4 | 21 | | 52.5 |
| 67 | 4 | 2 | 4 | 2 | 4 | 2 | 4 | 2 | 4 | 3 | 29 | | 72.5 |
| 68 | 3 | 2 | 4 | 2 | 4 | 2 | 4 | 2 | 4 | 3 | 28 | | 70 |
| 69 | 4 | 2 | 4 | 3 | 4 | 3 | 3 | 2 | 4 | 4 | 25 | | 62.5 |
| 70 | 3 | 2 | 4 | 2 | 3 | 3 | 4 | 2 | 4 | 2 | 27 | | 67.5 |
| 71 | 3 | 2 | 4 | 3 | 3 | 4 | 3 | 3 | 4 | 4 | 21 | | 52.5 |
| 72 | 5 | 1 | 5 | 1 | 5 | 3 | 2 | 2 | 4 | 2 | 32 | | 80 |
| 73 | 3 | 2 | 4 | 1 | 3 | 3 | 3 | 3 | 4 | 4 | 24 | | 60 |
| 74 | 4 | 3 | 3 | 3 | 4 | 4 | 4 | 2 | 4 | 4 | 23 | | 57.5 |
| 75 | 3 | 2 | 4 | 2 | 4 | 3 | 4 | 2 | 4 | 4 | 26 | | 65 |
| 76 | 3 | 3 | 3 | 3 | 4 | 3 | 3 | 3 | 3 | 4 | 20 | | 50 |
| Total SUS | | | | | | | | | | | | 64.14 | |
